# Foreground Segmentation-Based Density Grading Networks for Crowd Counting

**DOI:** 10.3390/s23198177

**Published:** 2023-09-29

**Authors:** Zelong Liu, Xin Zhou, Tao Zhou, Yuanyuan Chen

**Affiliations:** 1 College of Computer Science, Sichuan University, Chengdu 610000, China; 2020223045178@stu.scu.edu.cn (Z.L.); xinzhou@scu.edu.cn (X.Z.); 2 School of Automation Engineering, University of Electronic Science and Technology of China, Chengdu 610054, China; zhou_tao@uestc.edu.cn

**Keywords:** crowd counting, foreground segmentation, hierarchical foreground information, scale information

## Abstract

Estimating object counts within a single image or video frame represents a challenging yet pivotal task in the field of computer vision. Its increasing significance arises from its versatile applications across various domains, including public safety and urban planning. Among the various object counting tasks, crowd counting is particularly notable for its critical role in social security and urban planning. However, intricate backgrounds in images often lead to misidentifications, wherein the complex background is mistaken as the foreground, thereby inflating forecasting errors. Additionally, the uneven distribution of crowd density within the foreground further exacerbates predictive errors of the network. This paper introduces a novel architecture with a three-branch structure aimed at synergistically incorporating hierarchical foreground information and global scale information into density map estimation, thereby achieving more precise counting results. Hierarchical foreground information guides the network to perform distinct operations on regions with varying densities, while global scale information evaluates the overall density level of the image and adjusts the model’s global predictions accordingly. We also systematically investigate and compare three potential locations for integrating hierarchical foreground information into the density estimation network, ultimately determining the most effective placement.Through extensive comparative experiments across three datasets, we demonstrate the superior performance of our proposed method.

## 1. Introduction

The objective of crowd counting is to automatically analyze densely populated scenes, aiming to determine the precise count of individuals within raw images. This task has gained significant importance in the field of computer vision due to its potential applications in urban planning, crowd management, and extensive surveillance. Furthermore, networks designed for crowd counting can be applied to tasks with similar characteristics. Notable examples include vehicle detection and quantification at traffic intersections or along highways [1,2], estimation of wildlife group populations [3,4], automated enumeration of specific cells in microscopic images [5], and more.

Historically, methodologies for crowd counting can be broadly categorized into three main groups: detection-based methods [6,7,8], traditional regression-based approaches [9,10,11], and density map estimation-based techniques [12,13,14]. In cases where crowd density is relatively sparse, human body part detection through sliding windows has been employed for counting [15,16]. However, dense crowd scenarios introduce challenges such as occlusion and increased computational demands, making sole reliance on object detection inadequate for accurate counting. In such instances, traditional regression methods offer an alternative approach. This regression-based approach consists of two sequential phases: the initial extraction of low-level features and subsequent regression modeling. After capturing global and local features through various extraction techniques, distinct regression methodologies, including linear regression, piecewise linear regression [17], ridge regression [9], and neural networks, are employed to learn the mapping from these low-level features to crowd counts. Despite their utility, these techniques may overlook spatial information, leading to suboptimal predictive accuracy for crowd counts.

In recent years, the emergence of deep learning has effectively addressed the aforementioned issue. Deep neural networks inherently represent nonlinear functions, allowing for the establishment of complex and accurate mapping relationships as long as a large amount of training data is available. Leveraging the advantages of extensive datasets, the strategy of using neural networks to map images into density maps has gained widespread adoption for crowd counting. The density map emerges as a result of applying Gaussian processing to crowd labels, capturing not only the count of individuals but also crucial spatial and scale information related to the crowd’s distribution. By employing a neural network to transform input images into density maps, the inherent spatial information within the images can be fully utilized, ultimately leading to enhanced counting accuracy. A variety of deep neural network-based approaches have been introduced to tackle this problem, including cross-scene counting [12], MCNN [13], Switching CNN [18], ACSCP [19], ACSPNet [20], MMCNN [14] and others. However, crowd counting and density estimation tasks remain challenging, primarily due to the intricate elements present within the original images, such as complex buildings, roadways, and surrounding vegetation, which further compound the complexity of the crowd context.

**Figure 1:**
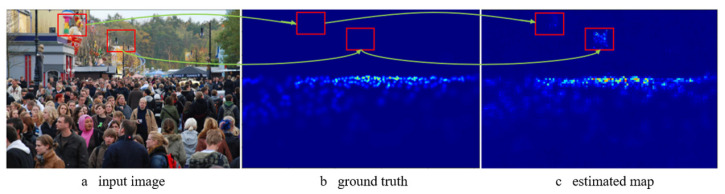
Examples of background misjudgments.

As discussed above, the mainstream method of crowd counting involves mapping the input image to a density map using a convolutional neural network and subsequently obtaining the count by integrating the density map. However, in these methods, the network often misclassifies the background (zero-valued area in the density map) as part of the head areas, leading to increased prediction errors, as highlighted by the red boxes in Figure 1. Additionally, the network tends to average high-density and low-density regions during computation, further contributing to the error. These errors occur for the following reasons. In the current density map labeling scheme, the values in the density map can have one of two states: a zero value means that there is no human head there, while a non-zero value means that there is a human head object there, and the value represents the local density. Density map estimation involves two steps: determining whether a pixel belongs to the foreground or background (object/non-object) and estimating the density value of the foreground region. However, when a single density map estimator is used for both tasks, the regressor may struggle to accurately distinguish between human heads and human head-like regions (such as grass and poorly lit forests), limited by the amount of data and network structure.

Motivated by these challenges, this paper introduces a novel network architecture that addresses these tasks sequentially. Specifically, after forecasting foreground/background regions, the proposed architecture hierarchically segregates foreground areas. Subsequently, it amalgamates hierarchical foreground information with intermediate density maps to derive the final density map. This strategy serves a dual purpose: it filters out human-like head regions in the background and effectively guides the network to make distinctive predictions for high-density and low-density regions, thereby enhancing the overall regression quality. The contributions of this paper can be summarized as follows.

- This paper proposes a novel architecture for crowd density estimation. To achieve accurate results, the model employs a three-branch structure that integrates foreground segmentation with density prediction and adjusts the global density using a scale factor, which evaluates the crowd density level in the original image.
- This article utilizes image segmentation techniques for crowd counting. Specifically, the image undergoes segmentation into three distinct categories: background, regions with low crowd density, and regions with high crowd density. This segmentation approach provides hierarchical foreground information that effectively guides the network in distinguishing areas with varying levels of crowd density.
- In this paper, we investigate and compare three distinct locations for the integration of hierarchical foreground information. Finally, we identify the hierarchical foreground information fusion location that yields the highest performance.

## 2. Related Work

### 2.1. Density Map-Based Methods

Benefiting from the rapid development of deep learning, contemporary crowd counting methods primarily rely on predicting density maps using deep neural networks. The density map-based approach for crowd counting was initially introduced in [1] aiming to incorporate spatial information into the regression process by learning a linear mapping between local patches and their corresponding density maps. However, mastering this linear mapping proved to be challenging. To mitigate this challenge, Pham et al. [21] employed random forest regression to map images to density maps, achieving satisfactory results by introducing a measure of crowdedness to train two different forests. While these traditional density map generation methods took spatial information into account, they relied solely on traditional hand-crafted features to extract low-level information and often struggled to produce high-quality density maps for more accurate counting. In recent years, due to the remarkable feature representation capabilities of CNNs, an increasing number of researchers have used CNN-based methods to enhance the accuracy of crowd density estimation. In this context, defining the original image as X and the network weights as θ, the process of crowd counting for a single image through a CNN can be expressed as follows: In this context, defining the original image as X and the network weights as θ, the process of crowd counting for a single image through a CNN can be expressed as Equation (1):

(1) $$C={\;\int \int \;}_{\sum }^{}F\left(X,\theta \right)dxdy$$

where F denotes the neural network. Wang et al. [22] developed an end-to-end CNN regression model to count the number of people in extremely crowded scenes. Their approach takes the entire image as input and directly outputs the final counts. In the same year, Zhang et al. [12] proposed a deep CNN method that alternately trained two related learning objectives: crowd density estimation and crowd count estimation. To address the dramatic changes in human head size caused by perspective, Zhang et al. [13] utilized a multi-column neural network (MCNN) to achieve more accurate counts by using differently sized receptive fields in each column. Sam et al. [18] introduced a path-switching architecture called Switching-CNN to handle crowd density changes within an image. Sindagi et al. [23] proposed a context pyramid CNN to combine different levels of context information. CSRNet [24] employed dilated convolution to expand the receptive field, improving accuracy and proving the effectiveness of dilated convolution. Xu et al. [25] introduced a novel approach to scale crowded patches, ensuring similar densities across all patches, addressing the issue of uneven density distribution. Recently, Zhou et al. [26] applied this method and achieved better results. Jiang et al. [27] incorporated an attention mechanism to enhance the prediction accuracy of their neural network. Additionally, some methods [28,29] have focused on designing new loss functions to effectively reduce estimation errors. Geng et al. [30] use high-level contextual information to improve the abilities of anchor-based detectors to detect dense and degenerate faces. This research served as a source of inspiration for our work. In addition, some recent works [26,31,32] have shown promising results and contributed to the advancement of crowd counting techniques.

### 2.2. Image Segmentation-Based Methods

Image segmentation is fundamental in computer vision and extensively applied to precise segmentation tasks, particularly in the domain of medical images. However, it is noteworthy that only a limited number of existing methods have leveraged image segmentation to aid crowd counting. MMCNN [33] is a multi-column multi-task network that incorporates the learning of location information based on MCNN. Although MMCNN imitated image segmentation, the loss function was still the Euclidean distance based on the density map, such that it could not accurately segment the crowd area. Hossain et al. [34] proposed to adjust the local regions in the image by means of a local attention network, an approach that also drew on the idea of image segmentation. However, there was no clear demarcation among the different density regions examined by this method. Unlike the methods discussed above, this paper explicitly performs foreground segmentation and obtains hierarchical foreground information to effectively guide the generation of the final density map.

## 3. Our Method

**Figure 2:**
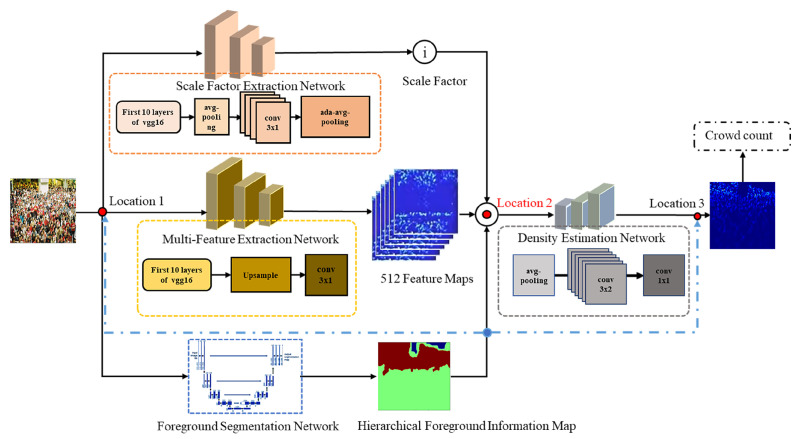
The architecture of the Foreground Segmentation-Based Density Grading Network. The three branches of the model are the scale factor extraction network, the density map generation network (consisting of a multi-feature extraction network and a density estimation network) and the foreground segmentation network, respectively.

To enhance counting accuracy by effectively integrating foreground information into the density map estimation process, we introduce our approach, named FSDGNet, whose architectural design is illustrated in Figure 2. FSDGNet employs a three-branch structure to combine hierarchical foreground information and global scale context into the density estimation network. The model comprises three branches: the density map generation network (comprising a multi-feature extraction network and a density estimation network), the foreground segmentation network, and the scale factor extraction network, respectively. The input image undergoes processing through the multi-feature extraction network, resulting in an intermediate density map. This intermediate density map is then combined with hierarchical foreground information using broadcasting operations and bitwise multiplication. The fusion position for the hierarchical foreground information, as described here, is located at position 2 in Figure 2. Detailed experimental information regarding the fusion position will be provided in Section 4.5.2. Finally, the results of the fusion operation are modified by the scale factors derived from the scale factor extraction network, producing the input of the density estimation network. The complete process can be outlined as Equation (2):

(2) M=D(X)∗B(F(X))∗G(X)

where X is the input image, while D, F, and G represent the multi-feature extraction network, foreground segmentation network, and scale factor extraction network, respectively. Moreover, B represents the broadcast operation and M is the input of the density estimation network that produces the final density map. In the following subsections, we will introduce the three branches of the model, methods used to generate the ground truth for the density map, and hierarchical foreground information.

### 3.1. Density Map Generation Network

The architecture of the density map generation network is composed of two main components: the multi-feature extraction network and the density estimation network. The initial 10 layers of the multi-feature extraction network replicate those found in the VGG16 network, such as in CSRNet. This design offers a dual advantage: it harnesses the powerful feature extraction capabilities inherent to the VGG model while preventing excessive network depth. Subsequently, an upsampling layer is added to the front-end network, scaling up the feature map, which is initially 1/8 the size of the original image, to 1/4 of the original image size. We up-sample here for two main reasons: first, to ensure the intermediate density map can be effectively fused with the hierarchical foreground information map, requiring them to be of the same size; and second, to enhance the resolution of the output density map for improved spatial accuracy. Finally, a convolution operation is applied to fuse the upsampled local features, resulting in the intermediate density map.

The density estimation network comprises an average pooling layer, six dilated convolutional layers, and an output layer. We substituted pooling layers with dilated convolutions to expand the receptive field while preserving resolution, thereby enhancing the preservation of spatial semantic information.

### 3.2. Scale Factor Extraction Network

The scale factor extraction network has an identical initial 10-layer structure as the multi-feature extraction network. Firstly, an average pooling layer is added after the base network, enhancing the network’s capability to comprehensively assess the global head density. Following this, four convolutional layers with a 3 × 1 kernel size are appended to adjust the output channel count to 1. After this sequence of convolutional operations, an adaptive average pooling operation is applied to the network output to quantize the feature map into a scale factor denoted as ’i’. Subsequently, the scale factor value is adjusted to fall within the range of 0 to 2 through the following operation:

(3) i=HardTan(i)+1

The density map produced by the multi-feature extraction network is modulated based on the scaling factor ’i’. Specifically, when the scale factor value exceeds 1, the output value of the multi-feature extraction network is increased from its initial value. Conversely, if the scale factor is less than 1, the output value is attenuated. If the scale factor is equal to 1, the output value remains unchanged. This adjustment mechanism ensures that the density map is properly scaled according to the scale factor.

**Figure 3:**
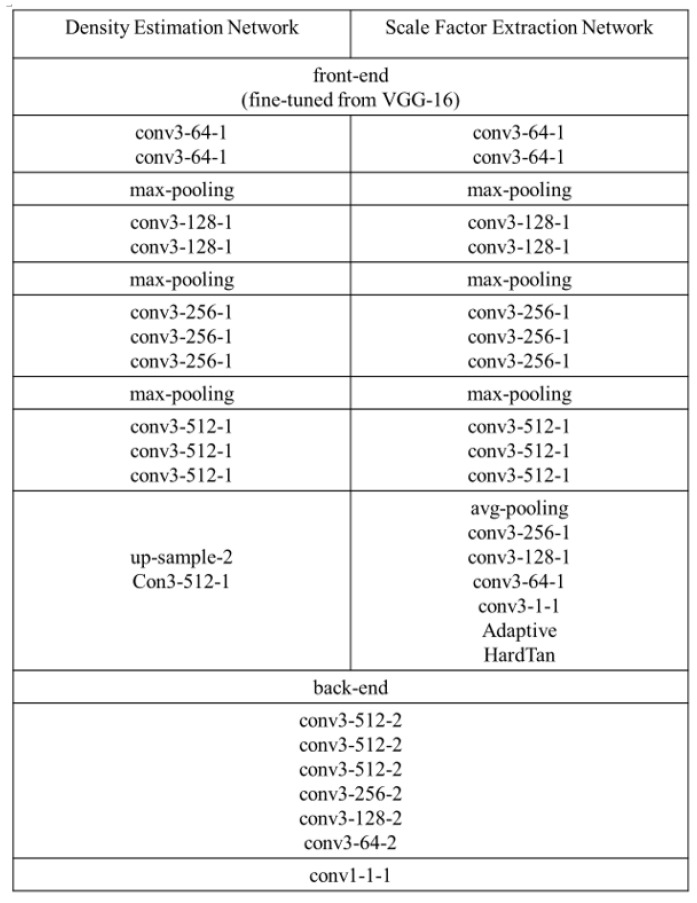
Structure of the density map generation network and scale factor extraction network.

The specific structures of the density estimation network and the scale factor extraction network are presented in Figure 3. The loss function used to train the network for density map generation is the Euclidean loss, which is widely employed in current practice.

### 3.3. Generation of Ground Truth and Foreground Segmentation Network

In this section, we will first introduce the method used to generate the Ground Truth map, which includes the density map and the hierarchical foreground information map. Following this, we will provide a detailed explanation of the foreground segmentation network in Section 3.3.3.

#### 3.3.1. Generation of Density Map

**Figure 4:**
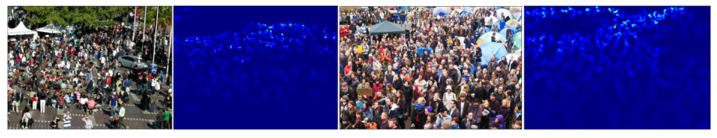
The original image and its corresponding density map.

This paper employs the density map generation method proposed in MCNN. An example density map is presented in Figure 4. A picture with N marked heads is represented by the following function:

(4) $$H\left(x\right)=\sum_{i=1}^{N}\delta \left(x-x_{i}\right)$$

This function represents the case where N heads are labeled in an image, where $\delta (x-x_{i})$ is the Dirac δ function which equals 1 at $\left(x-x_{i}\right)$ and 0 elsewhere. xi denotes the position of the i-th head. Then, this function is converted to a continuous density function by convolving it with a Gaussian function to generate a density map:

(5) $$F\left(x\right)=H\left(x\right)*G_{\sigma }\left(x\right)$$

The objective in generating the density map is to ascertain the parameters of the Gaussian function, particularly the diffusion parameter which dictates the size of the head on the density map. In this paper, the diffusion parameters for each person are adaptively determined based on the average distance between each person and their four nearest neighbor heads. For a head Xi in a given picture, we define its nearest k distances as: $d_{1}^{i},d_{2}^{i},\ldots ,d_{k}^{i}$. Consequently, the average distance can be computed as Equation (6):

(6) $$\bar{d^{i}}=\frac{1}{m}\sum_{j=1}^{m}d_{j}^{i}$$

Finally, we convolve the pulse function with the Gaussian kernel function to generate the density map. The exact density function is as Equation (7):

(7) $$F\left(x\right)=\sum_{i=1}^{N}\delta \left(x-x_{i}\right)*G_{\sigma_{i}}\left(x\right),\sigma_{i}=\beta \bar{d^{i}}$$

#### 3.3.2. Generation of Hierarchical Foreground Information Map

We then proceed with further processing of the density map obtained in Section 3.3.1 to create the hierarchical foreground information map. This process is essentially assigning a grade to each pixel within the density map.

**Figure 5:**
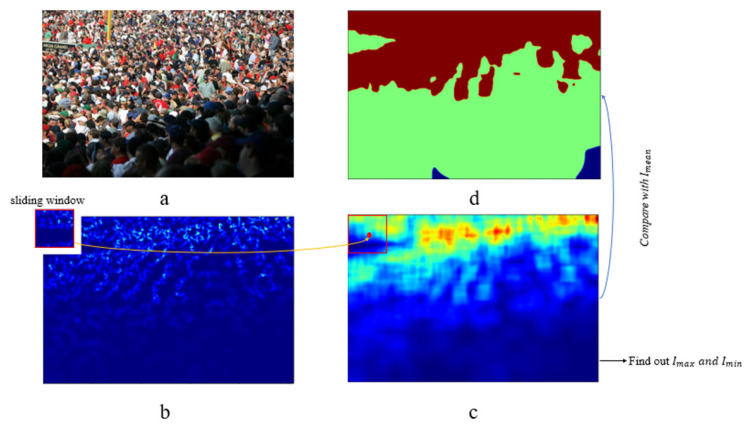
The generation process of hierarchical foreground information map. Image (**a**) is the original image, image (**b**) is the GT map, image (**c**) is the neighborhood density map and image (**d**) is the hierarchical foreground information map.

The procedure for generating the hierarchical foreground information map is illustrated in Figure 5. The process begins with the creation of a GT map “b” from an original image “a”, both with dimensions M∗N. This creation process is detailed in Section 3.3.1. Then, we scan the GT map “b” pixel by pixel employing a sliding window of dimensions 64∗64. This scanning enables the computation of the summation of pixel values within the local vicinity of each pixel, resulting in a neighborhood density map “c”. At this point, the segmentation between the background and foreground is complete. We now proceed with further processing to divide the foreground in neighborhood density map ’c’ into high-density and low-density areas. Continuing the process, density grading is applied to the foreground in “c.”While traversing the neighborhood density map pixel by pixel, we identify the largest pixel value, denoted as $I_{max}$, and the smallest non-zero pixel value, denoted as $I_{min}$. The threshold for density grading is subsequently set as the average value of these two values:

(8) $$I_{threshold}=\left(I_{max}+I_{min}\right)/2$$

Concluding this process, the neighborhood density map is once again traversed pixel by pixel. During this traversal, non-zero pixels that fall below the threshold are categorized as belonging to low-density regions, while pixels exceeding the threshold are classified as high-density regions. Based on experimental findings, optimal network performance is attained when the value for low-density regions is established at 0.9 and the value for high-density regions is set at 1.1. The resultant hierarchical foreground information map is depicted in Figure 5.

#### 3.3.3. Foreground Segmentation Network

**Figure 6:**
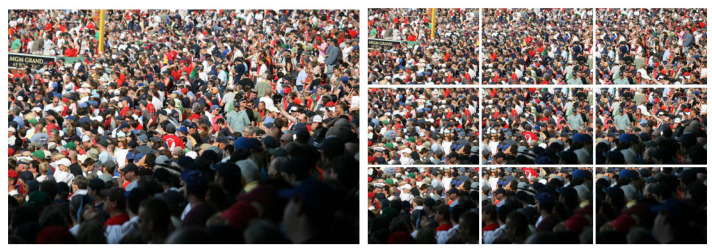
Training data of foreground segmentation network.

For the foreground segmentation component of our model, we have chosen the U-Net architecture, which is renowned for its exceptional segmentation efficacy. In contrast to the Euclidean distance used in the multi-task branch of MMCNN, we have opted for the cross-entropy function as the loss function for training the foreground segmentation network. Due to variations in dimensions among the original images in the Part A ShanghaiTech dataset, which is used for training, and considering the limited dataset size, we have employed a strategy of dividing each image into nine sub-images. Subsequently, each sub-image is resized to dimensions divisible by 16. This resizing ensures consistency in output dimensions with the graded mask GT map. The transformation of images before and after cropping is illustrated in Figure 6.

### 3.4. Multi-Scale Feature Extraction Module

As introduced in Section, the initial ten layers of the multi-feature extraction network adopt the corresponding layers from the VGG16 architecture, maintaining consistency with the baseline model. However, this structure fails to capture multi-scale information. To overcome this limitation, we have developed a multi-scale feature extraction module to replace a portion of the original network structure within the multi-feature extraction network. To be specific, we will replace the first three layers of the multi-feature extraction network with a multi-scale feature extraction module during the experiments. The comparative results of these experiments will be presented in Section 4.1.

**Figure 7:**
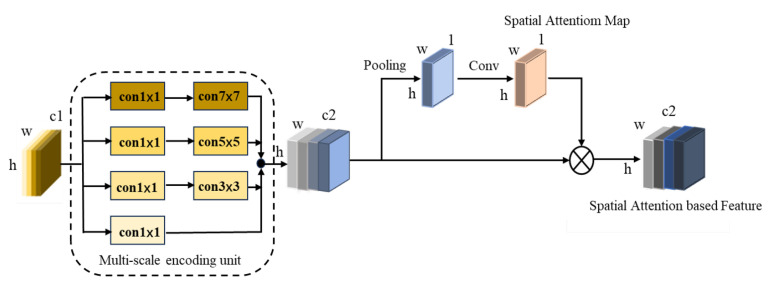
Multi-scale feature extraction module.

Figure 7 illustrates the structure of the multi-scale feature extraction module, which includes both a coding unit and a spatial soft attention unit. As depicted on the left side of Figure 7, the multi-scale encoding unit is designed to enhance the extraction of features relevant to densely populated crowd scenarios while simultaneously capturing the spatial specifics of crowd distribution. This is achieved by employing convolution kernels of various scales—1, 3, and 7—within the multi-scale encoding units. The design of the multi-scale encoding units empowers them to effectively capture features across different scales, effectively addressing challenges posed by occlusion and scale variation within crowd counting contexts. However, the feature map obtained at this time contains both location information and irrelevant background information. To reduce redundancy and enhance the network’s focus on essential information, a spatial attention unit is added after the multi-scale encoding unit. This unit serves to filter out extraneous details and heightens the network’s attention to important information regions.

## 4. Experiments

We evaluate our proposed model using three distinct datasets: ShanghaiTech, UCF_CC_50, and WorldExpo’10. Additionally, we conduct a comparative analysis of our approach against other crowd density estimation models, with the aim of demonstrating the effectiveness of our proposed method. Next, we will provide details about the datasets used in our experiments and outline the evaluation criteria applied. This will be followed by a comprehensive description of our experimental procedures.

### 4.1. Dataset

1. UCF_CC_50 is the first challenging dataset created from publicly available web images. It contains various densities and different perspective distortions in various scenes, including concerts, protests, stadiums, and marathons. Given the dataset’s size of only 50 images, it is typically processed with five-fold data partitioning before training and further processed with cross-validation during training. The limited dataset size often leads to suboptimal results, even with state-of-the-art CNN-based methods.
2. WorldExpo’10 is a large-scale, data-driven, cross-scenario crowd-counting dataset collected from the 2010 Shanghai World Expo. It comprises 1132 annotated video sequences captured by 108 surveillance cameras. The dataset contains a total of 3920 frames with a size of 576 × 720 pixels, encompassing scenes both indoors and outdoors, and includes annotations for 199,923 people.
3. ShanghaiTech is one of the largest crowd-counting datasets to emerge in recent years, comprising 1198 images and 330,165 annotations. The dataset is divided into two parts, A and B, based on differing density distributions. Part A consists of pictures randomly selected from the Internet, while those in part B are captured on a busy street in downtown Shanghai, featuring lower image density. Notably, the scale changes and perspective distortions within this dataset introduce new challenges and opportunities for many CNN-based network designs.

### 4.2. Experimental Details

The experiments are conducted using the PyTorch framework. We generate the ground-truth map of a density map using the geometric adaptive kernel proposed in Section 3.3.1. We then utilize this density map to generate the ground-truth map for the hierarchical foreground information map, which is used to train the foreground segmentation network.

We utilize the U-Net network as the foreground segmentation network. The initial 10 layers of both the density estimation network and the scale factor extraction network are initialized with pre-trained network parameters, while the other layers in the network architecture are initialized with normal distributions. We adopt mini-batch stochastic gradient descent (SGD) as the optimizer. The learning rate is initially set to 1 × 10 ^−5^ and is reduced by a factor of 0.1 every 20 epochs. The cross-entropy loss function is used for the foreground segmentation network, while the density estimation network employs the Euclidean loss function.

### 4.3. Evaluation Metrics

The two metrics we use in our experiments are Mean Absolute Error (MAE) and Mean Squared Error (MSE), which are defined as follows:

(9) $$MAE=\frac{1}{N}\sum_{i=1}^{N}\left|C_{I_{i}}^{pred}-C_{I_{i}}^{gt}\right|$$

(10) $$MSE=\frac{1}{N}\sqrt{\sum_{i=1}^{N}{\left|C_{I_{i}}^{pred}-C_{I_{i}}^{gt}\right|}^{2}}$$

N is the number of pictures, $C_{I_{i}}^{pred}$ is the predicted value and $C_{I_{i}}^{gt}$ is the true value.MAE assesses the accuracy of estimation results, while MSE evaluates the robustness of estimation. MSE is particularly sensitive to larger errors, making it more focused on identifying outliers or anomalies.

### 4.4. Comparison with Other Methods

In this section, we compare the proposed method with other methods on the ShanghaiTech, UCF_CC_50 and WorldExpo’10 datasets to demonstrate the effectiveness of our algorithm.

The comparative networks include MCNN, MANET, CSRNET, CPCNN, SANET, TEDNET, DENET and KDMG. We denote the network in which the first three layers of the density estimation network are replaced by the multi-scale feature extraction module as ’OURS_M’ in Table 1. The experimental results presented in Table 1 were obtained using our re-implemented code. One notable observation is that our network consistently achieves superior performance across all three datasets, outperforming the competing networks. It is worth highlighting that the performance of ’OURS_M’ is significantly better than that of ’OURS,’ demonstrating the effectiveness of the designed multi-scale feature extraction module in improving counting accuracy.

The reason for our network’s outperformance compared to MANET, another network grounded in image segmentation principles, can be attributed to our incorporation of graded foreground information. Prior to merging foreground information, the estimations in certain regions exceed the labeled counts, while the opposite is true for other areas. The overall error value is an average of these two conditions. When foreground information only focuses exclusively on discerning crowd areas, the mask leads to a reduction in the predicted head counts, resulting in decreased errors in regions where predicted counts surpass the labeled count. However, errors are exacerbated in areas where the predicted count falls short of the labeled count.

**Figure 8:**
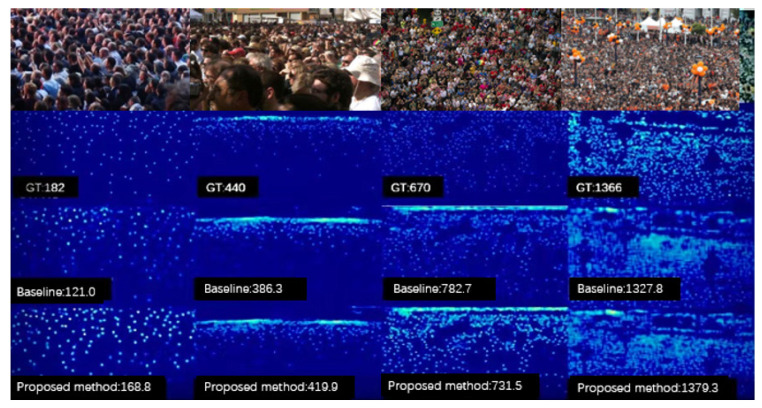
Some experimental results.

In contrast, the layered foreground infographic (with values of 0, 0.9, and 1.1 for different density areas) achieves balanced results by reducing the number of people in some areas while increasing the number of people in others. Figure 8 shows some experimental results of our proposed model and the baseline model on the dataset.

### 4.5. Ablation Experiment

In this section, we conduct a two-part investigation. First, we explore the impact of sliding window size on hierarchical foreground information maps in Section 4.5.1. Subsequently, in Section 4.5.2, we elaborate on the optimal fusion position for integrating the hierarchical foreground information map into the density map generation network. Both experiments were performed on the ShanghaiTech dataset.

#### 4.5.1. Study on Sliding Window Sizes

As depicted in Figure 5, during the process of hierarchical foreground information extraction, a fixed-size sliding window is employed to scan the ground truth density map pixel by pixel. The scanning operation can calculate neighborhood pixel values, thereby aiding in the grading of specific areas.

**Figure 9:**
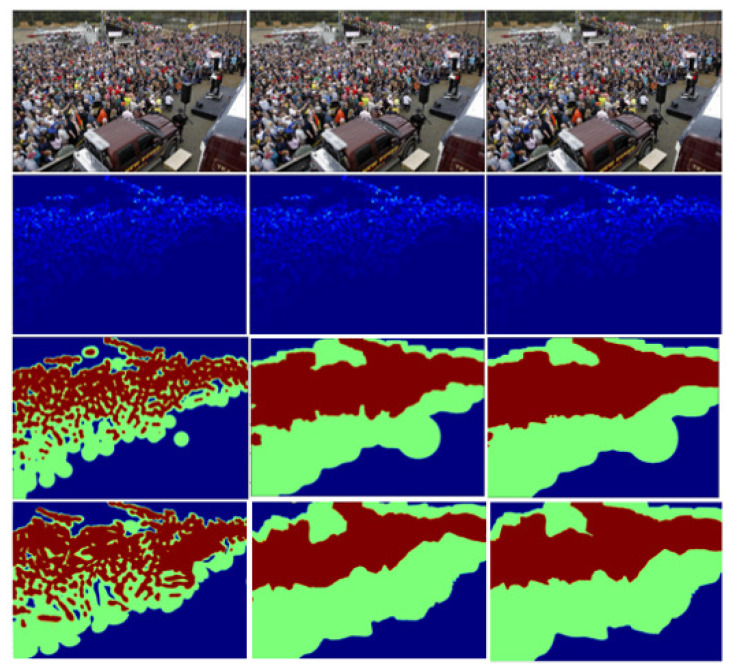
The results of the foreground segmentation network trained with the three differently sized windows. The sliding window sizes of the first, second, and third column are 48, 80, and 64, respectively. The first row is the input image, the second row is the ground truth of the density map, the third row is the predicted foreground information map, and the fourth row is the ground truth of the foreground information map.

The size of the sliding window is the neighborhood size of a single pixel, which affects the accuracy of the hierarchical foreground information generated by the foreground segmentation network. In the conducted experiments, we performed a series of comparative analyses by employing sliding windows with dimensions of 48, 64, and 80. These sliding windows of varying sizes are employed to generate ground truth maps for hierarchical foreground information, which in turn serve as training data for the foreground segmentation network. Figure 9 depicts the results of training the foreground segmentation network using these three different window sizes. Notably, when the window size is set to 64, the hierarchical foreground information map produced by the network aligns most closely with the ground truth map.

The experimental outcomes, succinctly outlined in Table 2, offer a comprehensive view of the accuracy performance achieved by employing three distinct sliding window sizes. Notably, the results underscore that the network attains its optimal performance when the sliding window size is configured at 64.

#### 4.5.2. Study on the Location of Hierarchical Foreground Information Fusion

In previous crowd density prediction research, distinguishing intricate backgrounds, such as trees and lawns, from actual crowd areas during density estimation, was a prevalent challenge. Our initial proposal aimed to utilize foreground information to guide crowd density estimation, enabling the density map generation network to adapt distinct approaches for regions with varying density levels and effectively mitigating prediction errors. For further study, we fuse hierarchical foreground information maps at three different locations, as shown in Figure 3. Simultaneously, we conduct a comparative experiment without the hierarchical foreground information map. Indeed, the integration of the hierarchical foreground information map at location 1 involves a pre-processing operation on the input of the density prediction network. The integration at at location 2 is to fuse the hierarchical foreground information with the feature information in the intermediate density map. Additionally, the fusion of the hierarchical foreground information map at location 3 is intended as a post-processing step for the output of the density estimation network. Essentially, this intricate approach systematically explores the integration method of the hierarchical foreground information at distinct stages of the density prediction process, thereby providing a nuanced understanding of its influence on network performance.

**Figure 10:**
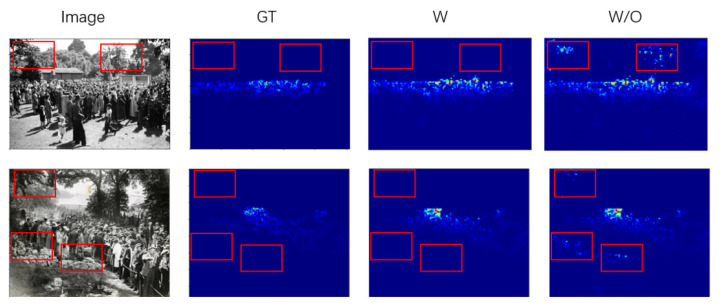
Different prediction results of partial images before and after removing the foreground segmetation network.

The comprehensive experimental findings are tabulated in Table 3, where it is evident that the most favorable outcomes are achieved by incorporating the hierarchical foreground information map at location 2. This can be attributed to the fact that our hierarchical foreground information map operates in a manner that dynamically adjusts the density estimation network’s attention across distinct regions, transcending a simplistic mask operation. Consequently, location 1 and location 3, which are more sensitive to confidence levels, do not exhibit the same level of effectiveness as location 2. Consequently, we opt to integrate the hierarchical foreground information map at location 2. To illustrate the effect of the hierarchical foreground information map in our experiments, we present the prediction results under the hierarchical foreground information map fusion condition (added at location 2), which is denoted as “W”, and the result without the hierarchical foreground information map, denoted as “W/O”. Figure 10 shows the different prediction results of partial images before and after removing the foreground segmentation network.

## 5. Conclusions

This paper addresses the challenge of crowd counting by harnessing deep neural networks. Our main contribution is to propose a novel architecture adopting a three-branch structure; this design effectively integrates hierarchical foreground information and global scale information into the density map estimation process, improving the accuracy of crowd counting. In addition, we demonstrate the effectiveness of the designed multi-scale feature extraction module for improving the counting accuracy through experiments. Finally, we systematically explore the methods for incorporating hierarchical foreground information into the density map generation network and identify the best-performing solutions. Through experimental validation, we demonstrate that the proposed method is effective and achieves a competitive crowd counting performance on various datasets.

## Figures and Tables

**Table 1:** Comparison of the results of different methods.

	SHTech Part A		SHTech Part B		UCF_CC_50		WorldExpo10					
Method	MAE	MSE	MAE	MSE	MAE	MSE	S1	S2	S3	S4	S5	avg
MCNN [13]	110.2	173.2	26.4	41.3	377.6	509.1	3.4	20.6	12.9	13	8.1	11.6
CPCNN [23]	73.6	110.2	20.1	30.1	295.8	320.9	2.9	14.7	10.5	10.9	5	8.8
CSRNET [24]	68.2	115	10.6	16.8	266.1	397.5	2.9	11.5	8.6	16.6	3.4	8.6
MANET [35]	66.3	109.4	16.9	28.4	283.3	411.6	3	16.7	11.6	12.5	4.1	9.6
SANet [36]	67	104.5	8.4	13.6	258.4	334.6	2.6	13.2	9.0	13.3	3.0	8.2
TEDNET [37]	64.2	109.1	8.2	12.7	249.4	354.6	2.3	10.1	11.3	13.8	2.6	8.0
DENET [38]	65.9	105.4	9.8	15.4	247.3	350.6	3.1	10.7	8.6	15.2	3.5	8.2
KDMG [39]	63.8	99.3	7.8	12.7	241.3	351.6	3.0	9.1	8.6	11.2	3.1	7.0
Ours	63.8	104.6	9.6	13.7	246.6	356.1	3.3	9.8	9.1	12.7	3.2	7.6
Ours_M	61.9	103.8	9.0	12.3	240.1	351.1	3.1	10.7	8.9	12.1	3.1	7.6

**Table 2:** Comparison of the results of using three sliding windows with different sizes.

Window Size	Without	48	64	80
MAE (SHTech PartA)	68.2	66.1	**63.8**	65.9
MSE (SHTech PartA)	115.3	107.6	**104.6**	109.4
MAE (SHTech PartB)	10.6	10.8	**9.6**	10.7
MSE (SHTech PartB)	16.8	14.0	**13.7**	15.9

The bold font indicates the results with the minimum error in each row.

**Table 3:** Comparison of the results of fusing hierarchical foreground information map at different locations.

Fusing Location	Without	Location 1	Location 2	Location 3
MAE (SHTech PartA)	68.2	66.1	**63.8**	66.6
MSE (SHTech PartA)	115.3	108.4	**104.6**	109.8
MAE (SHTech PartB)	10.6	10.8	**9.6**	10.7
MSE (SHTech PartB)	16.8	**13.5**	13.7	14.0

The bold font indicates the results with the minimum error in each row.

## Data Availability

We used publicly available datasets.

## References

[1] Ammar A., Koubaa A., Ahmed M., Saad A., Benjdira B. (2021). Vehicle Detection from Aerial Images Using Deep Learning: A Comparative Study. Electronics.

[2] Zhang H., Kyaw Z., Chang S.F., Chua T.S. Visual translation embedding network for visual relation detection. Proceedings of the IEEE Conference on Computer Vision and Pattern Recognition.

[3] Norouzzadeh M.S., Nguyen A., Kosmala M., Swanson A., Palmer M.S., Packer C., Clune J. (2018). Automatically identifying, counting, and describing wild animals in camera-trap images with deep learning. Proc. Natl. Acad. Sci. USA.

[4] Arteta C., Lempitsky V., Zisserman A. (2016). Counting in the wild. Proceedings of the European Conference on Computer Vision.

[5] Moradi Rad R., Saeedi P., Au J., Havelock J. (2019). Cell-Net: Embryonic Cell Counting and Centroid Localization via Residual Incremental Atrous Pyramid and Progressive Upsampling Convolution. IEEE Access.

[6] Dollar P., Wojek C., Schiele B., Perona P. (2012). Pedestrian Detection: An Evaluation of the State of the Art. IEEE Trans. Pattern Anal. Mach. Intell..

[7] Li M., Zhang Z., Huang K., Tan T. Estimating the number of people in crowded scenes by MID based foreground segmentation and head-shoulder detection. Proceedings of the 2008 19th International Conference on Pattern Recognition.

[8] Tuzel O., Porikli F., Meer P. (2008). Pedestrian Detection via Classification on Riemannian Manifolds. IEEE Trans. Pattern Anal. Mach. Intell..

[9] Chen K., Loy C.C., Gong S., Xiang T. Feature mining for localised crowd counting. Proceedings of the BMVC.

[10] Idrees H., Saleemi I., Seibert C., Shah M. Multi-source Multi-scale Counting in Extremely Dense Crowd Images. Proceedings of the IEEE Conference on Computer Vision and Pattern Recognition (CVPR).

[11] Rodriguez M., Laptev I., Sivic J., Audibert J.Y. Density-aware person detection and tracking in crowds. Proceedings of the 2011 International Conference on Computer Vision.

[12] Zhang C., Li H., Wang X., Yang X. Cross-scene crowd counting via deep convolutional neural networks. Proceedings of the 2015 IEEE Conference on Computer Vision and Pattern Recognition (CVPR).

[13] Zhang Y., Zhou D., Chen S., Gao S., Ma Y. Single-Image Crowd Counting via Multi-Column Convolutional Neural Network. Proceedings of the 2016 IEEE Conference on Computer Vision and Pattern Recognition (CVPR).

[14] Yang B., Cao J., Wang N., Zhang Y., Zou L. (2018). Counting challenging crowds robustly using a multi-column multi-task convolutional neural network. Signal Process. Image Commun..

[15] Subburaman V.B., Descamps A., Carincotte C. Counting People in the Crowd Using a Generic Head Detector. Proceedings of the 2012 IEEE Ninth International Conference on Advanced Video and Signal-Based Surveillance.

[16] Viola, Jones, Snow Detecting pedestrians using patterns of motion and appearance. Proceedings of the Ninth IEEE International Conference on Computer Vision.

[17] Chan A.B., Liang Z.S.J., Vasconcelos N. Privacy preserving crowd monitoring: Counting people without people models or tracking. Proceedings of the 2008 IEEE Conference on Computer Vision and Pattern Recognition.

[18] Sam D.B., Surya S., Babu R.V. Switching Convolutional Neural Network for Crowd Counting. Proceedings of the 2017 IEEE Conference on Computer Vision and Pattern Recognition (CVPR).

[19] Shen Z., Xu Y., Ni B., Wang M., Hu J., Yang X. Crowd Counting via Adversarial Cross-Scale Consistency Pursuit. Proceedings of the 2018 IEEE/CVF Conference on Computer Vision and Pattern Recognition.

[20] Ma J., Dai Y., Tan Y.P. (2019). Atrous convolutions spatial pyramid network for crowd counting and density estimation. Neurocomputing.

[21] Pham V.Q., Kozakaya T., Yamaguchi O., Okada R. COUNT Forest: CO-Voting Uncertain Number of Targets Using Random Forest for Crowd Density Estimation. Proceedings of the IEEE International Conference on Computer Vision (ICCV).

[22] Wang C., Zhang H., Yang L., Liu S., Cao X. Deep People Counting in Extremely Dense Crowds. Proceedings of the 23rd ACM International Conference on Multimedia (MM ’15).

[23] Sindagi V.A., Patel V.M. Generating High-Quality Crowd Density Maps Using Contextual Pyramid CNNs. Proceedings of the IEEE International Conference on Computer Vision (ICCV).

[24] Li Y., Zhang X., Chen D. Csrnet: Dilated convolutional neural networks for understanding the highly congested scenes. Proceedings of the IEEE conference on computer vision and pattern recognition.

[25] Xu C., Qiu K., Fu J., Bai S., Xu Y., Bai X. Learn to scale: Generating multipolar normalized density maps for crowd counting. Proceedings of the IEEE/CVF International Conference on Computer Vision.

[26] Zhou J.T., Zhang L., Jiawei D., Peng X., Fang Z., Xiao Z., Zhu H. (2021). Locality-aware crowd counting. IEEE Trans. Pattern Anal. Mach. Intell..

[27] Jiang X., Zhang L., Xu M., Zhang T., Lv P., Zhou B., Yang X., Pang Y. Attention Scaling for Crowd Counting. Proceedings of the IEEE/CVF Conference on Computer Vision and Pattern Recognition (CVPR).

[28] Ma Z., Wei X., Hong X., Gong Y. Bayesian loss for crowd count estimation with point supervision. Proceedings of the IEEE/CVF International Conference on Computer Vision.

[29] Wan J., Liu Z., Chan A.B. A Generalized Loss Function for Crowd Counting and Localization. Proceedings of the IEEE/CVF Conference on Computer Vision and Pattern Recognition (CVPR).

[30] Geng Q., Liang D., Zhou H., Zhang L., Sun H., Liu N. (2021). Dense Face Detection via High-level Context Mining. Proceedings of the 2021 16th IEEE International Conference on Automatic Face and Gesture Recognition (FG 2021).

[31] Liu X., Yang J., Ding W., Wang T., Wang Z., Xiong J. (2020). Adaptive mixture regression network with local counting map for crowd counting. Proceedings of the European Conference on Computer Vision.

[32] Zhang Y., Zhao H., Duan Z., Huang L., Deng J., Zhang Q. (2021). Congested Crowd Counting via Adaptive Multi-Scale Context Learning. Sensors.

[33] Hatamizadeh A., Tang Y., Nath V., Yang D., Myronenko A., Landman B., Roth H.R., Xu D. UNETR: Transformers for 3D Medical Image Segmentation. Proceedings of the IEEE/CVF Winter Conference on Applications of Computer Vision (WACV).

[34] Hossain M., Hosseinzadeh M., Chanda O., Wang Y. Crowd Counting Using Scale-Aware Attention Networks. Proceedings of the 2019 IEEE Winter Conference on Applications of Computer Vision (WACV).

[35] Jiang S., Lu X., Lei Y., Liu L. (2019). Mask-aware networks for crowd counting. IEEE Trans. Circuits Syst. Video Technol..

[36] Cao X., Wang Z., Zhao Y., Su F. Scale Aggregation Network for Accurate and Efficient Crowd Counting. Proceedings of the European Conference on Computer Vision (ECCV).

[37] Jiang X., Xiao Z., Zhang B., Zhen X., Cao X., Doermann D., Shao L. Crowd counting and density estimation by trellis encoder-decoder networks. Proceedings of the IEEE/CVF conference on computer vision and pattern recognition.

[38] Liu L., Jiang J., Jia W., Amirgholipour S., Wang Y., Zeibots M., He X. (2020). Denet: A universal network for counting crowd with varying densities and scales. IEEE Trans. Multimed..

[39] Wan J., Wang Q., Chan A.B. (2022). Kernel-Based Density Map Generation for Dense Object Counting. IEEE Trans. Pattern Anal. Mach. Intell..

